# Old and New Precipitants in Hepatic Encephalopathy: A New Look at a Field in Continuous Evolution

**DOI:** 10.3390/jcm12031187

**Published:** 2023-02-02

**Authors:** Daniele Bellafante, Stefania Gioia, Jessica Faccioli, Oliviero Riggio, Lorenzo Ridola, Silvia Nardelli

**Affiliations:** Department of Translational and Precision Medicine, Sapienza University of Rome, 00185 Roma, Italy

**Keywords:** overt hepatic encephalopathy, minimal hepatic encephalopathy, sarcopenia, portosystemic shunts, ammonia, liver cirrhosis

## Abstract

Hepatic encephalopathy (HE) is a common complication in patients with advanced liver disease. It is a brain dysfunction characterized by neurological and psychiatric symptoms that significantly affects quality of life, morbidity and mortality of patients. HE has various precipitants that can potentially promote its onset, alone or in combination. Among the historically well-known precipitants, such as infections, gastrointestinal bleeding, dehydration, electrolyte disorders and constipation, recent studies have highlighted the role of malnutrition and portosystemic shunts as new precipitating factors of HE. The identification, management and correction of these factors are fundamental for effective HE treatment, in addition to pharmacological therapy with non-absorbable disaccharides and/or antibiotics.

## 1. Hepatic Encephalopathy

The most common complication of advanced liver disease is hepatic encephalopathy (HE) [1]. HE is a particular kind of brain dysfunction which is typical of liver cirrhosis; it is characterized by nonspecific neurological and psychiatric manifestations [2], ranging from subclinical psychometric alterations to delirium and coma [3]. Covert HE includes minimal HE (MHE), characterized by abnormalities on neuropsychological, neurophysiological or psychometric tests (PHES, CFF, S-ANT1, CCHE), and a mild clinical form (HE grade I) in which abnormalities are usually recognized by caregivers and physicians. Overt HE (OHE) includes clinically detectable neurological and psychiatric disorders, such as asterixis, spatial and temporal disorientation, somnolence and, in the most severe form, delirium and coma (HE grade IV) [2]. It has been proposed that HE be classified as follows based on the underlying disease:

Type A: hepatic encephalopathy due to of acute liver failure
Type B: hepatic encephalopathy due to portal-systemic bypass with no intrinsic hepatocellular disease
Type C: hepatic encephalopathy due to cirrhosis with portal hypertension or systemic shunting [2]. Type C HE is the most common form, and this review is focused on it. It is not easy to establish the incidence of HE in patients with liver disease due to the use of different clinical tools to diagnose HE and the fact that there are a lot of studies in which covert and overt HE are not well distinguished [4,5]. The prevalence of covert HE ranges between 20% and 80% among cirrhotic patients [4,6]. The prevalence of overt HE at the time of the first diagnosis of cirrhosis is between 10% and 20% [4]. In decompensated cirrhosis, the prevalence of overt HE ranges between 16% and 21% [1]. The overall incidence of overt HE after Transjugular Intrahepatic Portosystemic Shunt (TIPS) ranges between 25% and 45%, but if only new onset or worsening HE are considered, a lower percentage of HE was found [7]. HE is the complication of advanced liver disease that most frequently leads to hospitalization [8].

The pathogenesis of HE is not yet fully understood. High ammonia levels in the blood flow play a central role in the development of HE [9]. Ammonia is predominantly produced in the gut as a product of protein digestion, amino acid deamination and bacterial urease activity. In addition, ammonia is produced and utilized in other organs, like muscles, kidneys and brain, in various biochemical reactions (i.e., the amidation of glutamate in muscles consumes ammonia) [2,8]. In liver failure, the main pathway of ammonia metabolism is compromised, leading to hyperammonemia [10]. Moreover, in patients with large portosystemic shunts, this mechanism is altered because blood flow from the gut, overloaded with ammonia, skips the liver, transporting higher levels of ammonia into the systemic circulation. High concentrations of ammonia can pass through blood–brain barrier, causing astrocyte swelling and cerebral edema [11]. Even if it is known that ammonia is crucial in the development of HE, a direct correlation between levels of ammonia and the grade of HE has not been confirmed by studies [12]. This suggests the existence of other pathogenetic mechanisms, such as systemic inflammation and intestinal barrier alterations [13,14]. In the setting of intestinal barrier dysfunction and systemic inflammation, gut flora and its by-products such as ammonia, indoles, oxindoles and endotoxin play important roles in the pathogenesis of HE [13]. It has been demonstrated that gut microbiota differ between cirrhotic patients and healthy controls. The fecal microbiota of patients with cirrhosis is characterized by a higher prevalence of Alcaligeneceae, Porphyromonadaceae and Enterobacteriaceae, species that are strongly associated with cognition and inflammation in HE [15]. This study indicates that future trials with targeted prebiotics and probiotics and/or fecal transplantation should aim at enhancing cognition through the modulation of these microbiome components [16,17].

The diagnosis of HE is based on clinical features (asterixis, temporal and spatial disorientation, somnolence, changes of personality, stupor and finally, coma) and on the exclusion of other neurological disorders that may imitate HE, such as dementia, meningoencephalitis, hypercapnia, electrolyte alterations, psychosis, drug or alcohol intoxication, Wilson disease and brain masses. Once the diagnosis is made, every effort should be made to identify single or multiple precipitants and implement appropriate corrective measures [2].

## 2. “Classical” Precipitants and Their Management

### 2.1. Infections 

Infections are one of the most frequent causes of worsening liver function in patients with acute or chronic liver disease, increasing mortality, morbidity and the prevalence of other complications [18]. Bacterial infections are a major cause of gastrointestinal bleeding, kidney failure, acute-on-chronic-liver failure (ACLF) and hepatic encephalopathy (HE) in patients with liver disease [19]. Patients with cirrhosis are more likely to develop infections [20] because of multiple factors, i.e., a certain grade of immuno-dysfunction [21], increased translocation of intestinal bacteria into the bloodstream [22] and the alteration of gut microbiota in terms of a reduction of beneficial phyla and an overgrowth of pathogenic phyla [23]. The prevalence of bacterial infections in patients hospitalized for decompensated cirrhosis is around 25–46% [24,25]. Patients with higher Child-Pugh and MELD scores and with ascites or gastrointestinal bleeding are more susceptible to infections [19]. Spontaneous bacterial peritonitis (SBP) represents the most common infection in these patients, followed by urinary tract infections, pneumonia, bloodstream infections and skin and soft tissue infections [24,25,26]. Historically, gram negative bacteria (notably *Enterobacteriaceae*) are most commonly responsible for SBP and urinary tract infections, while gram positive are a major cause of pneumonia [25]. Nowadays, the epidemiology of infections in cirrhotic patients has changed due to the use of quinolones for the prophylaxis of bacterial infections. This has led to a higher number of gram positive infections, a larger use of third generation cephalosporins, which has increased bacterial resistance, and an increase of the prevalence of multidrug-resistant and extensively drug resistant bacterial infections [20]. Bacterial resistance leads to a higher number of cases of health-care associated (HCA) and hospital-acquired (HA) infections. Merli et al. demonstrated that HCA and HA are the most frequent infections in hospitalized cirrhotic patients [27], and that infections are related to increased cognitive impairment in patients with cirrhosis compared to those without liver disease [28]. Bacterial products cause an excessive pro-inflammatory response and the production of pro-inflammatory molecules, leading to immunopathological tissue damage [29]. Additionally, cell necrosis induced by infections leads to the release into the bloodstream of danger-associated molecular patterns (DAMPs) that stimulate innate immune system and pro-inflammatory responses [29]. Regarding brain damage, the immunological response to infections may result in cerebral edema in patients with cirrhosis, which is a substrate of the development of HE [19]. Alabsawy et al. wanted to determine if overt HE predisposes patients to the onset of infections; they demonstrated that overt HE is an independent risk factor for new infections in cirrhotic patients with acute decompensation [30]. These findings suggest the presence of a mutual relationship between HE and infections, in which one predisposes patients to the other. This finding should stimulate new research to identify pathophysiological and possible target therapies [31].

As shown in Table 1, every patient admitted to hospital with signs of cirrhosis decompensation should be tested for potential infections [32]. A chest X-ray should be performed in order to exclude the presence of compatible parenchymal infiltrates suggestive of pneumonia. Additionally, urine analysis and, when necessary, urinecolture should be executed to identify urinary tract infections. SBP should be investigated in patients with ascites. A diagnostic paracentesis is mandatory upon admission in hospital; a diagnosis of SBP is made if the polymorphonuclear cell (PMN, also referred to as neutrophils) count in the ascitic fluid is ≥250 cells/mm^3^, the culture results are positive and secondary causes of peritonitis have been excluded. If systemic infection is suspected, blood cultures should be performed [32]. As suggested by the EASL guidelines, empirical antibiotic therapy has to be started promptly if an infection is suspected, and the choice of antibiotics should be guided by the environment (community vs. nosocomial vs. health-care associated infection), local antibiotic resistances and the severity and type of infection [33].

The implementation of preventive measures for infections is crucial in these patients. To this end, the use of vaccinations should be encouraged (not only the mandatory ones, but also recommended vaccinations, according to the guidelines in different countries), and good general hygiene and dental status are strongly recommended.

### 2.2. Gastrointestinal Bleeding

Gastrointestinal bleeding (variceal or non-variceal bleeding) is one of the most common precipitants of HE. Nowadays, the relationship between gastrointestinal bleeding and HE is well-known. Gastrointestinal bleeding leads to an increase in blood ammonia [8]; the incidence of HE after a GI bleeding ranges from 10 to 39% in high-risk patients (Child Pugh C or B with active bleeding). Rattanasupar et al. tried to define clinical predictors of HE in cirrhotic patients presenting with acute variceal bleeding [34]; according to their retrospective study, Child-Pugh score C, serum levels of potassium < 3 mEq/L, white blood cells count > 10,000/mm^3^ and Hb < 8 g/dL were considered significant predictors of the development of HE. Moreover, cirrhotic patients who develop HE have higher morbidity and mortality rates [34]. According to the latest RCTs, there are only sparse and conflicting data about prophylaxis of HE in patients with acute gastrointestinal bleeding. Two recent single-center randomized studies demonstrated that the use of lactulose in cirrhotic patients admitted to hospital with acute variceal and non-variceal gastrointestinal bleeding is effective in preventing HE compared to a placebo [35,36]. Another recent RCT suggested that primary prophylaxis with anti-ammonium drugs (lactulose, L-ornithine L-aspartate or rifaximin) proved to be effective in preventing HE in patients with variceal bleeding compared to a placebo [37]. In contrast, in a more recent randomized trial, Rattanasapur et al. showed that the use of primary prophylaxis with lactulose in cirrhotic patients with acute upper gastrointestinal bleeding was ineffective compared to placebo to prevent HE, and unnecessary treatment with laxatives should be avoided [38]. These conflicting data should stimulate new studies on the effectiveness of primary prophylaxis in these patients. According to the latest EASL guidelines, in cirrhotic patients with GI bleeding, rapid removal of blood from the gastrointestinal tract (with mannitol or lactulose by nasogastric tube or lactulose enema) is an effective treatment to prevent HE [3].

According to the latest Baveno VII guidelines, in patients presenting with suspected acute variceal bleeding, vasoactive drugs and antibiotic prophylaxis should be started as soon as possible, and upper endoscopy should be performed within 12 h of presentation [39]. Once it has been determined if bleeding is occurring in the esophageal or gastric varices, endoscopic therapy should be performed. In case of esophageal variceal bleeding, endoscopic band ligation is recommended; tissue adhesives (N-butyl-cyanoacrylate/thrombin) are recommended in gastro-esophageal varices type 2 and in isolated gastric varices. Both endoscopic therapies can be used in gastro-esophageal varices type 1 [39]. Additionally, TIPS is indicated in selected cases [40]. To prevent recurrent variceal hemorrhage, the first-line prophylactic therapy is the combination of traditional non-selective beta-blockers (NSBBs) or carvedilol and endoscopic ligation [39]. TIPS can also be used in secondary prophylaxis of variceal bleeding in patients who rebleed despite traditional NSBBs or carvedilol and endoscopic ligation [40]. In acute variceal bleeding which is refractory to traditional pharmacological and endoscopic therapies, balloon tamponade or self-expandable metal stents should be used as a bridge therapy before a more definite treatment, such as TIPS [39,41], is implemented (Table 1).

To prevent the first episode of GI bleeding, treatment with NSBBs should be considered in patients with clinically significant portal hypertension (high-risk varices). There is no evidence that endoscopic therapies might prevent ascites or HE, but in patients who have contraindications or intolerance to NSBBs, endoscopic band ligation is recommended as a primary prophylaxis [39].

### 2.3. Dehydratation

Dehydration is another precipitant of HE in patients with advanced liver disease. There are multiple causes of dehydration in patients with cirrhosis, such as the overuse of diuretics, large volume paracentesis and lactulose-related diarrhea in patients treated for prophylaxis for HE [42]. Dehydration can also be caused by comorbidities that patients may have, like diabetes mellitus [43]; therefore, dehydration is often a multifactorial condition. Pantham et al. demonstrated that dehydration is the most recognized precipitant for overt HE in patients with cirrhosis [42]. In particular, a comparative study by Bajaj et al. showed that lactulose-associated dehydration was associated with recurrent HE episodes [44]. In dehydrated patients, it is important to remove the cause of dehydration where possible (stop diuretics when necessary), give supportive therapies to restore idro-electrolytic values and, when necessary, properly titrate lactulose in order to maintain its safety profile (Table 1).

### 2.4. Electrolytes Alterations

Hyponatremia is one of the most common electrolyte alterations observed in cirrhotic patients [45]. Sodium serum levels < 135 mmol/L are associated with high rates of complications in liver cirrhosis, such as ascites, renal failure, sepsis, HE and mortality [46]. Hyponatremia in cirrhotic patients is due to a combination of pathophysiological mechanisms, including splanchnic vasodilatation and increased secretion of antidiuretic-hormone (ADH) [45]. Cirrhotic patients have a pathological reduction of brain organic osmolytes (which are responsible for the compensatory osmoregulatory mechanism against brain cells swelling during hyponatremia). Therefore, in case of hyponatremia, this brain compensatory osmoregulatory mechanism is enhanced; this may be relevant to the pathogenesis of HE and neurological symptoms in cirrhotic patients with hyponatremia [47]. Guevara et al. discovered that low levels of serum sodium are a major risk factor of development of overt HE in cirrhotic patients [48] and, in particular, in patients with refractory ascites [49]. The debate about whether hyponatremia causes a form of brain disturbance that is separate from HE or causes HE directly is still open. Watson et al. suggested that there is an interdependence of liver failure and hyponatremia, and that the improvement of hyponatremia in patients with cirrhosis leads to an increase in the speed of complex information processing analyzed with the trail-making test (TMT) [50]. Bossen et al. demonstrated an association between serum sodium and HE incidence; the hazard rate of HE development increase by 8% for every mmol/L decrease in serum sodium [51]. Additionally, hypokalemia (potassium < 3 mEq/L) is a precipitant of HE, but a less relevant one than hyponatremia [42,52]. In cases of hypovolemic hyponatremia, the administration of normal saline is sufficient, while in cases of hypervolemic hyponatremia, fluid restriction (<1000 mL/day) or hypertonic saline or albumine administration is suggested (Table 1).

### 2.5. Constipation

Another important precipitant of the development of HE is constipation [2,53]. Constipation leads to an increased orocecal transit time, a higher reabsorption of toxic metabolites from the gut and a higher level of blood ammonia. A longer orocecal transit time is associated with an increased risk of developing HE [54]. The role of constipation in the origin of hepatic encephalopathy is still controversial, both because it is a parameter that can hardly be objectified and because it often coexists with other precipitants (e.g., infections, dehydration), making it difficult to identify the real cause of HE. Management of constipation consists of using osmotic oral laxatives and/or cathartics and bowel enemas (Table 1). In particular, lactulose and lactitol are the first line therapy, both because they reduce the absorption of ammonium from the colon, reducing its circulating levels, and because they have a cathartic action, clearing the gut of ammonia before it can be absorbed, thereby contributing to the improvement of HE. Lactitol seems to be more tolerable and produces fewer side effects than to lactulose. The goal of therapy is to ensure two or three bowel movements a day in the absence of diarrhea or dehydration [2].

### 2.6. Others

The consumption of alcohol in patients with advanced liver disease is also a major risk factor for the development of HE, so any patient with a diagnosis of cirrhosis must stop alcohol consumption.

Limited evidence is available to support the hypothesis that benzodiazepines (BDZ) increase the risk of HE in patients with cirrhosis. A study by Grønbæk L et al. demonstrated that cirrhotic patients who had begun using BDZ had a markedly increased risk of developing first-time HE in the 3rd to the 10th day after starting BZD therapy, while in the 1st and 2nd and after 28 days, there was no such an excess risk [55].

## 3. New Precipitants and Their Management

### 3.1. Muscle Alterations

Muscle alterations are frequent in patients with liver cirrhosis and include the loss of muscle mass (sarcopenia) and the infiltration of muscle mass by intermuscular and intramuscular fat (myosteatosis) [56]. Both conditions are frequent in patients with chronic liver disease [57]; in fact, sarcopenia is observed in up to 70% of cirrhotic patients, and it is associated to a higher mortality in these patients [58,59]. A recent study showed that the accuracy of the MELD score in predicting mortality at 3 and 6 months could be ameliorated by considering muscle alterations [60]. In cirrhotic patients, muscle alterations can occur for multiple reasons, i.e., inadequate dietary intake, impaired absorption and substrate utilization due to liver disease. In normal conditions, the liver is involved in ammonia detoxification among its various other functions; this mechanism is altered in patients with cirrhosis and/or in the presence of porto-systemic shunts. Skeletal muscle has a compensatory function in ammonia metabolism and clearance, so a reduction in the quantity of muscle mass leads to an increase of blood ammonia levels, enhancing the risk of HE episodes in cirrhotic patients [57,61]. It has been demonstrated that muscle alterations are strong risk factors for the development of both minimal and overt HE [61,62]. According to the results of Nardelli et al. [61], myosteatosis was detected in 68% and sarcopenia in 84% of patients with overt HE. Moreover, both myosteatosis (62.5% versus 12.5%, *p* < 0.001) and sarcopenia (84% versus 31%, *p* < 0.001) were more frequent in patients with MHE than in those without. In that study, it was demonstrated that survival was significantly lower in malnourished patients [61]. The relationship between HE and sarcopenia is now well-established; the co-presence of sarcopenia and a previous history of HE is associated with higher mortality [63]. Since malnutrition worsens the prognosis, in addition to the treatment of the underlying causes of cirrhosis and its complications when they occur, an early assessment of nutritional status should be performed in these patients [64]. Nowadays, both instrumental and non-instrumental tools are available to asses nutritional status in cirrhotic patients, such as the bioimpedance test, handgrip strength, DEXA and computed tomography scans for the first category, and food diaries, objective examinations, biochemical parameters (level of total plasma protein), anthropometric measurements, the creatine-to-weight ratio and subjective global assessment questionnaires in the latter category [65]. As well as nutritional assessments, dietary control is a valid tool to improve nutritional status and prognosis. According to recent guidelines, diet in cirrhotic patients with and without HE should not differ; a daily caloric intake in non-obese cirrhotic patient of 30–40 kcal/Kg/day with a protein intake of 1–1.5 g/Kg/day and a diet that is rich in vegetable and dairy proteins are recommended [66,67]. Electrolyte and vitamin monitoring should be done, and these must be supplemented in case of deficiency [65]. Dietary protein restriction in patients with HE is no longer recommended [67,68,69]. Physical exercise, as long it increases muscle mass, may reduce the risk of HE episodes [65,70].

### 3.2. Spontaneous Portosystemic Shunts (SPSS)

In patients with advanced liver disease, the increase of portal hypertension can lead to the opening of communications between portal veins and systemic circulation. These communications are called spontaneous portosystemic shunts (SPSS) [71]. Patients with cirrhosis frequently develop SPSS as a complication of long-term portal hypertension [39]. SPSS can be divided into “left-sided” if they are located to the left of the spleno-porto-mesenteric confluence and “right-sided” if they are located to the right [72]. Splenorenal shunts (“left-sided”) are most associated with the development of recurrent or persistent HE [73]. Blood flow diversion directly into the systemic circulation, due to the presence of these shunts, causes an increase in the ammonia levels in the circulation, increasing the risk of HE. A retrospective multicenter study by Simon-Taléro et al. showed that the prevalence and size of shunts increase with deterioration of liver function, and the risk of HE and portal hypertension complications is associated with the presence of SPSS [74]. Furthermore, Riggio et al. demonstrated a higher prevalence of SPSS in cirrhotic patients with chronic HE compared to those without [75]. A recent multicenter study investigated the variables independently associated with the presence of SPSS (as detected by CT scan) and found that portal vein thrombosis and cirrhosis Child-Pugh C were independently associated with SPSS of any size, while previous HE and portal vein thrombosis were the only variables associated with the presence of large size SPSS (>1 cm) [76]. Patients with advanced chronic liver disease are more likely to have more than 1 SPSS. A multicenter study by Praktiknjo et al. demonstrated that the total cross-sectional SPSS area (TSA) (rather than diameter of the single largest SPSS) predicts survival. It has been suggested that a TSA > 83 mm^2^ increases the risk of OHE [77].

In case of refractory or persistent HE due to the presence of SPSS, the elective treatment is shunt obliteration. Several studies have demonstrated the efficacy and safety of this treatment, revealing improved survival, a reduction of HE episodes, amelioration of symptoms and a reduction of the number of hospitalizations [78,79,80]. The two main indications of shunt embolization are bleeding from gastric varices and treatment of persistent/refractory HE. The oldest therapeutic technique is balloon-occluded retrograde transvenous obliteration (BRTO), in which a balloon catheter is placed retrogradely into the shunt to stop the communication between portal and systemic circulation; then, a sclerosant is injected into the shunt and into the varices for thrombus formation. The newest techniques are plug-assisted retrograde transvenous obliteration (PARTO) and coil-assisted retrograde transvenous obliteration (CARTO). In PARTO, a vascular plug is used instead of a balloon catheter [81]; large size coils are used in CARTO [82].

### 3.3. Transjugular Intrahepatic Portosystemic Shunt (TIPS)

A transjugular intrahepatic portosystemic shunt (TIPS) is an interventional radiological procedure that consists of the creation of an artificial channel of communication between the portal vein and the hepatic veins via the insertion of an intrahepatic stent [39,40]. TIPS is used to treat complications associated with portal hypertension, such as variceal bleeding and refractory ascites [40]. One of the major complications of TIPS is post-TIPS HE [83]. The incidence of new or worsening HE after TIPS placement is 35–50% [84,85,86], and the risk of refractory HE after TIPS is 8% [87]. Nowadays, there is little evidence for the existence of an effective pharmacological treatment to prevent the onset of post-TIPS HE. Riggio et al. demonstrated that there was no difference in HE incidence in the first month after TIPS in three groups of patients (a placebo group, a lactitol group and a rifaximin group), although that study had a small sample size and a short duration of follow-up after TIPS [88]. A more recent RCT by Bureau et al. compared rifaximin to placebo for the prevention of post-TIPS HE. According to the results, the cumulative incidence of HE in all the enrolled patients was lower in the rifaximin group compared to the placebo group. Otherwise, if only patients without history of HE were considered, the trend remained positive for the rifaximin group, although statistical significance among the two groups was not achieved [89]. According to the latest EASL guidelines, in patients with cirrhosis and history of overt HE, rifaximin can be considered for post-TIPS HE prophylaxis in cases of non-urgent TIPS placement, but further studies are needed to support the efficacy of this [3]. There is also a correlation between incidence of post-TIPS HE and stent diameter [87]. A lower portosystemic pressure gradient is associated with a higher risk of HE. A recent RCT compared 8 mm stents vs. 10 mm stents in patients who underwent TIPS placement to prevent variceal re-bleeding; it emerged that while 10 mm and 8 mm showed similar shunt function, the risk of HE was halved with 8 mm stents. Therefore, the latter should be used for the prevention of variceal bleeding in patients with advanced liver disease [90]. The introduction of new stents with controlled expansion may allow the operator to control the portosystemic pressure gradient, thereby reducing the risk of post-TIPS HE [91]. The use of under-dilated TIPS (stent caliber < 8 mm) seemed to be feasible and was associated with lower rates of HE, with no increase in recurrent variceal hemorrhage and ascites, compared to normal caliber TIPS [92]. Numerous studies have shown that TIPS is associated with improvement in sarcopenia, so the risk of post-TIPS HE might gradually decrease with skeletal muscle growth after TIPS placement [93,94]. In addition, TIPS plays an important role in controlling symptoms such as gastrointestinal bleeding or SBP, which are precipitants of HE. Therefore, the high risk of HE might exist only in the short-term post-TIPS period, while the long-term risk of HE in patients treated with TIPS might not be higher than that in patients treated with other methods. Further studies are needed to investigate these differences. Nevertheless, given the high incidence of post-TIPS HE and the lack of effective and standardized treatments to prevent HE, the selection of patients to undergo TIPS implantation remains crucial, and risk factors (older age, previous HE episodes, impaired liver function, impaired renal function, malnutrition) should be considered.

## 4. Overview of Available Pharmacological Treatments for HE

The management of overt HE is based on the identification of HE precipitants and their correction and on empirical treatment aimed at reducing blood levels of ammonia [2]. In the previous sections, precipitants and their management were discussed. To date, the most common drugs used for HE treatment are non-absorbable disaccharides (lactulose or lactitol) and non-absorbable antibiotics (rifaximin). After the first episode of HE, secondary prophylaxis should be started with non-absorbable disaccharides at a dosage of 20 mL of syrup or the equivalent in granules twice daily. The dosage can be modulated in order to obtain 2–3 soft tools per day [2,3]. Rifaximin should be added if HE recurs (one or more HE episodes within 6 months) [3,95].

In the following section, we provide is an overview of treatments that may benefit patients with HE:

Probiotics: probiotics are live micro-organisms that are capable of modulating the host gut microbiome, decreasing the burden of pathogenic intestinal bacteria and ameliorating the toxic effects of bacterial translocation in cirrhosis [96]. There is a lack of strong evidence supporting the use of probiotics in patients with HE. A Cochrane review published in 2017 included 21 trials and more of 1400 patients comparing patients with HE and cirrhosis treated with probiotics vs. those treated with placebo, no intervention or any other treatment for HE. According to that review, probiotics improved the development of overt hepatic encephalopathy, quality of life and plasma ammonia concentrations. It should be emphasized that most trials suffered from a high risk of systematic and random errors, making the quality of the available evidence very low [97]. A more recent meta-analysis demonstrated that probiotics could decrease serum ammonia and endotoxin levels, improve MHE and prevent overt HE development in patients with liver cirrhosis [98]. However, due to the fact that the data on the effects of probiotics in HE are not consistent, currently, there are several ongoing studies seeking to clarify the role of probiotics on gut microbiota modulation in these patients.

Fecal microbiota transplantation (FMT): FMT is currently mainly used for recurrent infection of C. Difficile [99]. There is one case report and two small RCTs that demonstrate the efficacy of FMT in cirrhotic patients with HE in terms of reducing cognitive impairment and number of hospitalizations [100,101,102]. The rationale behind the use of FMT in cirrhotic patients with HE is to modulate gut microbiota composition, thereby reducing the gut dysbiosis which is typical of cirrhotic patients [17,103]. A recent review of eight studies showed long-term improvement in cognitive performance in cirrhotic patients who underwent FMT [104]. However, the clinical applicability of FMT for treating HE remains under investigation.

L-ornithine l-aspartate (LOLA): L-ornithine and L-aspartate are amino-acids involved in metabolic pathways that lead to the production of urea and glutamine, incorporating ammonia molecules. In this way, LOLA lowers blood ammonia [105]. A recent review with a meta-analysis showed that treatment with LOLA is effective as a secondary prophylaxis of overt HE and as a primary prophylaxis of HE following acute variceal bleeding compared to placebo or no treatment [106]. A RCT comparing LOLA, lactulose and probiotics showed similar efficacy [107]. A recent Cochrane review confirmed the efficacy of LOLA on mortality and amelioration of HE compared to placebo or no intervention. However, because of the low quality of evidence, these findings are subject to scrutiny. The evidence of a possible beneficial effect of L-ornithine L-aspartate on hepatic encephalopathy, when compared with probiotics, was also of very low quality, and no other benefits were demonstrated in comparison with other active agents [108]. A recent double-blind RCT by Arpan J et al. evaluated the role of intravenous LOLA in patients with cirrhosis with high grade HE (OHE grade III-IV). It was demonstrated that a combination of LOLA with lactulose and rifaximin was more effective than only lactulose and rifaximin in improving the grade of HE and recovery time from encephalopathy [109]. However, more data are needed to establish the role of LOLA in HE treatment.

Polyethylene glycol (PEG): PEG administrated by nasogastric tube alone or in association with lactulose has been associated with a more rapid resolution of overt HE requiring hospitalization [110]. Compared to lactulose, PEG can lead to a more rapid resolution of HE during the first 24 h and shorten the length of hospital stay without increasing the rate of adverse effects [111].

Other antibiotics: neomycin, metronidazole and vancomycin have been used but are currently not recommended because of their potential systemic toxicity [2].

Branched chain amino acids (BCAAs): BCAAs include valine, leucine and isoleucine. In cirrhotic patients, the availability of these amino acids is reduced, thus impairing the conversion of ammonia to glutamine in skeletal muscle. A Cochrane review (including 16 RCTs) comparing BCAAs vs. placebo/no intervention, diet, lactulose and neomycin showed that BCAAs had a beneficial effect on HE when trials with a lactulose or neomycin control were excluded, as well as no difference between those interventions when BCAAs and lactulose or neomycin were compared [112]. Additional studies are needed to compare BCAAs with other antibiotics and non-adsorbable disaccharides.

## 5. Conclusions

Hepatic encephalopathy is one of the main complications of advanced liver disease and portosystemic shunts. On the one hand, the treatment is based on the use of non-absorbable disaccharides and non-absorbable antibiotics. Data on the use of other agents, like LOLA, PEG and modulators of fecal microbiota (probiotics and FMT) are still incomplete, and more trials are needed to establish their efficacy. On the other hand, it is crucial to identify and correctly manage precipitants as soon as possible in order to ameliorate the neurological symptoms. When possible, it is important to start primary prophylaxis of HE. Secondary prophylaxis should always be started after the first episode of HE.

## Figures and Tables

**Table 1:** Checklist for the identification and treatment of precipitating factors for hepatic encephalopathy.

Precipitants	Diagnosis	Management
Infections	- Pneumonia: ask for recent history of fever or cough, leucocyte count and CRP, chest X-ray or HRCT scan - Urinary tract infections: ask for recent history of urinary symptoms, leucocyte count and CRP, urine test and urinoculture. - SBP: diagnostic paracentesis - Sepsis: ask for recent history of fever, leucocyte count, CRP and procalcitonin, blood culture - Acute gastroenteritis: diarrhea with or without fever and presence of pathogens in stool - Others: presence of pathogens	Start empirical antibiotic therapy promptly is infection is suspected, (the choice of antibiotics should be guided by the environment, local antibiotic resistances and the severity and type of infection). Once the antibiogram is available, start specific antibiotic therapy.
Gastrointestinal Bleeding	Any evidence of upper GI tract bleeding (hematemesis, melena)	Variceal Bleeding: stabilize the patient if unstable, start vasoactive drugs and antibiotic prophylaxis and perform upper endoscopy within 12 h: - EV: endoscopic band ligation - GOV2 and IGV1-2: adhesive tissues - GOV1: endoscopic band ligation or adhesive tissues. Consider TIPS placement in selected cases.Non-Variceal Bleeding: stabilize the patient if unstable + start PPI iv at high dosage + perform upper endoscopy within 24 h (<12 h if high risk patient) + endoscopic hemostasys if indicated.
Dehydration	Any sign of dehydration (skin and mucosal dryness, confused state) in a suitable context (patient with vomiting, diarrhea, diuretics abuse), as well as sodium, creatinine and hematocrit increase	Correct dehydration (fluid therapy, stop diuretics)
Electrolyte Disorders	Hyponatremia (sodium < 130 mEq/L);	- hypovolemic hyponatremia: administration of normal saline; - hypervolemic hyponatremia: fluid restriction (<1000 mL/day), consider hypertonic saline or albumine administration.
	Hypokaliemia (potassium < 3 mEq/L)	Administeroral or iv KCl
Constipation	More than 24 h without passing stool or demonstration of significant fecal retention in colon	Oral laxatives and/or cathartics, bowel enemas
Malnutrition	- Non-instrumental tools: BMI, food diary, objective examination, biochemical parameters, anthropometric measurements, creatinine-to-weight ratio, subjective global assessment questionnaire	Dietary advice for a correct supply of nutrients
	- Instrumental tools: bioimpedance test, handgrip strength test, DEXA, computed tomography scan	Physical exercise to improve muscle trophism based on the patient’s potential
Portosystemic shunts		
- Spontaneous	Evidence of SPSS at radiologic imaging (eco, CT scan or MRI)	Radiological shunt obliteration (BRTO, CARTO, PARTO) in case of recurrent/persistent HE
- TIPS	Anamnesis and/or evidence of TIPS at radiologic imaging	TIPS revision if necessary
Alcohol and Drugs	Anamnesis	Stop alcohol consumption and hepatotoxic drugs
